# How to Improve the Explanatory Power of an Intelligent Textbook: a Case Study in Legal Writing

**DOI:** 10.1007/s40593-024-00399-w

**Published:** 2024-05-06

**Authors:** Francesco Sovrano, Kevin Ashley, Peter Leonid Brusilovsky, Fabio Vitali

**Affiliations:** 1 Department of Informatics, University of Zurich, Rämistrasse 71, 8006, Zürich, Switzerland; 2 Department of Computer Science and Engineering, University of Bologna, Bologna, Italy; 3 School of Law, University of Pittsburgh, Pittsburgh, PA USA; 4 School of Computing and Information, University of Pittsburgh, Pittsburgh, PA USA

**Keywords:** Intelligent textbooks, Education, Explanatory artificial intelligence, Answer retrieval, Automated question extraction

## Abstract

Explanatory processes are at the core of scientific investigation, legal reasoning, and education. However, effectively explaining complex or large amounts of information, such as that contained in a textbook or library, in an intuitive, user-centered way is still an open challenge. Indeed, different people may search for and request different types of information, even though texts typically have a predefined exposition and content. With this paper, we investigate how explanatory AI can better exploit the full potential of the vast and rich content library at our disposal. Based on a recent theory of explanations from Ordinary Language Philosophy, which frames the explanation process as illocutionary question-answering, we have developed a new type of interactive and adaptive textbook. Using the latest question-answering technology, our e-book software (YAI4Edu, for short) generates on-demand, expandable explanations that can help readers effectively explore teaching materials in a pedagogically productive way. It does this by extracting a specialized knowledge graph from a collection of books or other resources that helps identify the most relevant questions to be answered for a satisfactory explanation. We tested our technology with excerpts from a textbook that teaches how to write legal memoranda in the U.S. legal system. Then, to see whether YAI4Edu-enhanced textbooks are better than random and existing, general-purpose explanatory tools, we conducted a within-subjects user study with more than 100 English-speaking students. The students rated YAI4Edu’s explanations the highest. According to the students, the explanatory content generated by YAI4Edu is, on average, statistically better than two baseline alternatives (*P* values below .005).

## Introduction

As pointed out by UNESCO, the United Nations specialized agency for education, in one of its recent publications (Miao et al., 2021), the opportunities and challenges that Artificial Intelligence (AI) offers for education in the AI era are yet to be fully understood. Nonetheless, the expectations are high given the potential of AI to foster our ability to acquire and convey knowledge. Indeed, since the invention of writing, we have collected a vast amount of written content that now forms the basis of our collective wisdom and industriousness.

However, fully harnessing the potential of such written knowledge by automatically explaining it in an intuitive and user-centered way is still an **open problem**. For instance, different explainees^1^ may search for and request different types of information, even though, usually, the exposition and content of an explanatory document (e.g., a book, an article, a web page, or technical documentation) are predetermined and static.

Different books on the same subject expound the same knowledge in different ways and with different levels of detail. Additionally, this type of static representation is sub-optimal and time-consuming in the most generic scenario because helpful information may be sparse and scattered over hundreds or thousands of pages. As a result, explaining to a person can be an extremely challenging task, regardless of whether the explanandum^2^ is from technical documentation, a scientific article, a regulation or a textbook. Additionally, the complexity of this task is increased by the elusiveness of the notion of explanation.

To address the problem of automatically explaining, we study how to automatically enhance static educational books by making them more interactive. We do this by reducing the sparsity of relevant information, thereby increasing the *explanatory power* of the medium while also linking it to a knowledge graph extracted from a collection of supplementary materials.

The approach is based on a recent theory from Ordinary Language Philosophy (Sovrano & Vitali, 2022a, explained in section “Explanations According to Ordinary Language Philosophy”), which views explaining as more than just providing answers to questions. It emphasizes that explaining involves answering unspoken or implicit questions in a meaningful and relevant way to the individual seeking understanding.

For instance, if you ask “How are you doing?” and the response is “I am fine”, this is not an explanation. However, if the response is “I am okay because I was worried I could have tested positive for COVID-19, but I am not, and [...]”, it becomes an explanation. The reason: this response intends to create an understanding of the person’s state of being.

This concept of responding to implicit questions is called *illocution* (Sovrano & Vitali, 2022a). It makes the explanation process more focused on the individual seeking understanding. By anticipating these implicit questions, both the person explaining and the person seeking understanding can communicate more efficiently, resulting in a reduction of steps required to explain something^3^.

Hence, assuming that the goal of an educational e-book is to explain something to the reader and consistent with the abovementioned definition of explanations (Sovrano & Vitali, 2022a, b), we built an explanatory process capable, through question-answering, of organizing a textbook’s explanatory space^4^ to enable a reader to more efficiently and effectively retrieve helpful information. Specifically, our **proposed solution** consists of a pipeline of Explanatory AI (YAI) algorithms and heuristics to build, on top of textbooks, intelligent interfaces for:

- answering explicit questions asked by a user;
- automatically identifying the topics that can be explained by the contents of a textbook (or any collection of texts);
- generating explanations by anticipating a set of useful (implicit) questions a user might have about the textbook.

Assuming that the content of a textbook (or a collection of texts integrating it) properly explains a given explanandum, our work relies on the following hypothesis:

### Hypothesis 1

The most useful implicit questions a user may have about a collection of texts are those best answered in the collection as a whole. These questions are neither too detailed (because they would otherwise only be answered in a minor part of the collection) nor too general (because they would be answered inaccurately in the more detailed textual content).

If the assumption mentioned above holds, we believe that the hypothesis is correct for the following reasons:

- any question falling outside the scope of the collection of documents could not be answered, thus not being useful;
- whoever wrote the textbook tried to explain as well as possible (for her/his narrative purposes) the most important topics at hand, thus (according to the adopted definition of explanation) implicitly identifying the most important questions whose answers provide a good overview of the topics.

Consequently, our **main contribution** is a novel pipeline of YAI software for enhancing the explanatory power of a textbook based on an algorithm (called Intelligent Explanation Generator) for identifying the questions best answered by a collection of texts. Indeed, according to theory, what makes a YAI good at explaining is its ability to identify implicit and relevant questions to answer, somehow anticipating the needs of the explainee.

**Fig. 1:**
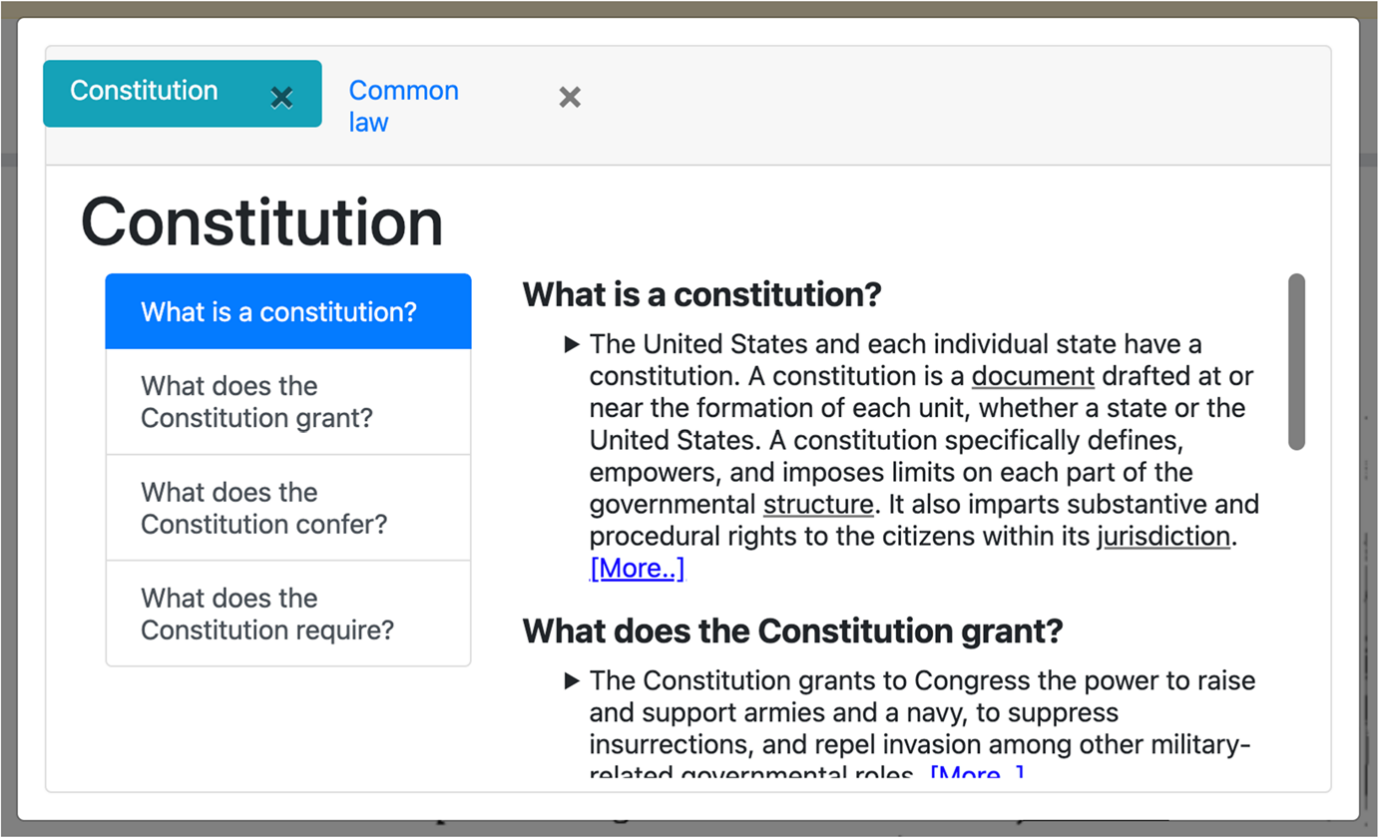
**Example of intelligent explanatory overview generated by YAI4Edu.** This figure contains an example of interactive *overview* in the form of a *scrollspy* showing relevant questions and answers as explanations. The reader can select a new topic to overview by clicking on any underlined word

As a **case study** for our proposed YAI for education (YAI4Edu, for short), we considered a teaching scenario where the excerpts of a textbook, *“United States Legal Language and Culture: An Introduction to the U.S. Common Law System”* (Brostoff & Sinsheimer, 2013), together with the encyclopedia of the *Legal Information Institute of the Cornell Law School* and thousands of cases of the Board of Veterans’ Appeals (BVA)^5^ are used for teaching how to write a legal memorandum^6^ in support of a disability claim for Post-Traumatic Stress Disorder (PTSD) according to the U.S. legal system.

The complexity of legal jargon and intricate language in the field of law offers a substantial test for YAI4Edu, making our case study an ideal showcase of its abilities. We utilize over 16,000 diverse resources for this study, including a textbook actively used in a University of Pittsburgh course. This enhances the study’s real-world practicality and educational relevance. When coupled with the important societal context of veterans’ PTSD disability claims, our case study highlights the potential of AI applications in education, particularly in complex fields such as law. For an example of an intelligent explanation generated by YAI4Edu in this scenario, see Fig. 1.

As an **example** to clarify what Hypothesis 1 means in this case, let us suppose we want to explain what a legal memorandum is. The textbook (Brostoff & Sinsheimer, 2013) does it by describing what a memo is in a legal sense, what it is for, what the proper form of a legal memorandum is, and what sections it should include. The textbook also provides *secondary details*, explaining each step of drafting a memorandum, why writing a memo is difficult, what the heading of a memorandum contains, and so on. Hence, in this case, Hypothesis 1 implies that the most useful implicit questions to ask are not those whose answers are only secondary details. This is because they are too specific to represent the whole textbook’s explanatory content adequately. Instead, the best choices are the main questions such as “what is the proper form of a legal memorandum”, “what is a memo in a legal sense”, because they best represent the content of the textbook.

To evaluate YAI4Edu and verify Hypothesis 1, we conducted a within-subjects **user study**, comprising more than 100 students. This was done to study how different strategies to identify helpful implicit questions impact the quality of the resulting explanations. In particular, during the study, different explanations (an example is shown in Fig. 5) were given to English-speaking students about the task of *writing a legal memorandum*. For more details about the explanations used in the experiment, see Table 2.

We compared the explanations generated by the Intelligent Explanation Generator (relying on Hypothesis 1) with those of the following two **baseline algorithms**:

- a **random explanations generator**: an algorithm that organizes explanations by *randomly* selecting implicit questions from those answered by the corpus of considered texts;
- a **generic explanations generator**: an algorithm that uses very *generic questions* (e.g., why, how, what) instead of those extracted from the textbook, under the assumption that all possible (implicit) questions are instances of such generic questions.

The **results** supported Hypothesis 1. They showed that our Intelligent Explanation Generator outperforms the baselines mentioned above in all the considered explanations, selecting questions that are not too specific or generic. The differences in performance were statistically significant (*P* values below .005). Notably, the results also show that the explanations generated from generic questions are significantly better than those generated from random questions, giving further evidence in support of the validity of the hypothesis.

**Synopsis.** In section “Related Work”, we discuss existing literature on interactive e-books for education, focusing on AI-based solutions. In section “Background”, we provide sufficient background to understand the theory behind the algorithms presented in section “YAI4Edu: a YAI for Improving the Explanatory Power of an Intelligent Textbook”. Then, in section “Case Study: A Textbook for Teaching How to Write Legal Memoranda”, we present our case study on legal writing, and in section “Experiment”, we introduce the experiment for verifying Hypothesis 1. Finally, in section “Discussion: Results and Limitations” and section “Limitations and Future Work”, we discuss the experimental results and the limitations of the proposed approach, while also highlighting potential avenues for future work.

## Related Work

Some studies suggest that using intelligent textbooks^7^ and interactive e-books leads to an increase in use, motivation and learning gains versus static e-books (Ericson, 2019). Of the several streams of work on the topic of interactive e-books and intelligent textbooks, most focus on the cognitive process of the reader, studying how to enhance the pedagogical productivity of textbooks through expert systems or sophisticated interfaces. They usually accomplish this by:

- showing personal progress through open learner models (Kay & Kummerfeld, 2019, 2021);
- specializing on ad hoc tasks through some domain modeling (Beier & Rau, 2022; Deligiannis et al., 2019; Chacon et al., 2021; Matsuda & Shimmei, 2019);
- modeling a student through questions, in order to identify and suggest personalized contents (Thaker et al., 2019; Mohammed & Shaffer, 2021; Matsuda & Shimmei, 2019);
- associating pedagogically valuable quizzes and exercises to portions of the e-book (Wang et al., 2022; Shimmei & Matsuda, 2022; Campenhout et al., 2021a, b);
- providing tools for manually creating new interactive e-books (Wang et al., 2021; Pursel et al., 2019; Kluga et al., 2019).

The use of AI for the automatic generation of interactive e-books seems to be under-explored. In one such project, (Barria-Pineda et al., 2022) propose to automatically augment the sections of existing books with related YouTube videos by directly annotating the PDF, thus without breaking the structure of these textbooks.

Unlike the previous literature examples, our approach attempts to fully automatically convert an existing e-book into an interactive version by exploiting theories of explanations, intelligent interfaces, and YAI. In particular, we are not interested in the task of generating verbatim^8^ questions for quizzes or exercises as in Wang et al. (2022); Shimmei and Matsuda (2022); Campenhout et al. (2021a, 2021b). Instead we pursue the idea that questions (even non-verbatim ones) can be a practical criterion to organize and categorize the content of explanations. Moreover, instead of considering any question as a suitable candidate for this task, we empirically show that some questions are more useful than others and that the best questions for explanatory overviews are neither too generic nor too specific.

## Background

This section aims to provide enough background information for the reader to understand what an explanation is according to Ordinary Language Philosophy and the consequent theory of YAI.

### Explanations According to Ordinary Language Philosophy

The concept of explanation in philosophy^9^ began to have a more precise role in the 20th century with the growth and development of the philosophy of science. The publication by Hempel and Oppenheim (1948) of their “Studies in the Logic of Explanation” gave rise to what is considered the first theory of explanations: the deductive-nomological model. Sometime later, this first model came to be considered fatally flawed (Bromberger, 1966; Salmon, 1984). Indeed, Hempel’s epistemic theory of explanations is not empiricist: it is concerned (mistakenly) only with logical form, so an explanation can be such regardless of the actual processes and entities conceptually required to understand it.

Several more modern and competing theories of explanation have resulted from this criticism (Mayes, 2001). Some of these theories, for instance, those proposed by Salmon (1984) and van Fraassen (1980), take a scientific approach, framing explanations purely as answers to why questions. These theories represent a theoretical and largely scientific interpretation of the process of explanation.

On the other hand, other theorists have taken a more grounded approach to the idea of explanations. These are grounded in the practicalities of how people perform explanations in everyday life (Mayes, 2001). An exemplar of this approach is the theory proposed by Achinstein (1983). His theory, which is firmly rooted in Ordinary Language Philosophy, underscores the communicative or linguistic aspect of an explanation. It highlights the importance of an explanation’s role in fostering understanding between individuals by answering questions.

In particular, according to Achinstein’s theory, explaining is an *illocutionary* act born of a clear intention to produce new understandings in an explainee by providing a correct content-giving answer to an open-ended question. According to this view, answering by “filling in the blanks” of a pre-defined answer template, as with most one-size-fits-all approaches, prevents answering from being explanatory since it lacks this *illocutionary* purpose.

Despite this definition, *illocution* seems too abstract to implement in an actual software application. Nonetheless, recent efforts towards the automated generation of explanations (Sovrano & Vitali, 2021, 2022b), have shown that it may be possible to define *illocution* in a more “computer-friendly” way. Indeed, as stated by Sovrano and Vitali (2021), illocution in explaining involves informed and *pertinent* answers not just to the main question but also to other questions that are implicitly relevant to the explanations and the explainee. These questions can be understood as instances of archetypes such as why, why not, how, what for, what if, what, who, when, where, how much, etc.

#### Definition 1

(Illocution in Explaining) Explaining is an illocutionary act that provides answers to an explicit question on some topic along with answers to several other implicit or unformulated questions deemed necessary for the explainee to understand the topic properly. Sometimes these implicit questions can be inferred through a thorough analysis of the explainee’s background knowledge, history, and objectives, also considering Frequently Asked Questions (FAQs). However, in the most generic case, no assumption can be made about the explainee’s knowledge and objectives. The only implicit questions that can then be exploited for *illocution* are the most generic ones, called *archetypal questions*.

For example, if someone asks “How are you doing?”, an answer like “I am good” would not be considered an explanation. By contrast, a different answer, such as “I am happy because I just got a paper accepted at this important venue, and [...]” would generally be considered an explanation because it answers other *archetypal questions* along with the main question.

#### Definition 2

(Archetypal Question) An *archetypal question* is an archetype applied on a specific aspect of the explanandum. Examples of archetypes are the interrogative particles (e.g., why, how, what, who, when, where), or their derivatives (e.g., why not, what for, what if, how much), or also more complex interrogative formulas (e.g., what reason, what cause, what effect). Accordingly, the same archetypal question may be rewritten in several different ways, as “why” can be rewritten in “what is the reason” or “what is the cause”.

### Archetypal Questions in Linguistic Theories

Casting the semantic annotations of individual propositions as narrating an archetypal question-answer pair recently gained increasing attention in computational linguistics (He et al., 2015; FitzGerald et al., 2018; Michael et al., 2018; Pyatkin et al., 2020). In particular, the main *archetypes* coming from Abstract Meaning Representation theory (Michael et al., 2018) are: what, who, how, where, when, which, whose, why. We refer to these archetypes as the *primary* ones because they consist only of interrogative particles.

On the other hand, the main *archetypes* coming from PDTB-style discourse theory (Pyatkin et al., 2020) (also called *secondary archetypes* because they make use of the *primary archetypes*) are: in what manner, what is the reason, what is the result, what is an example, after what, while what, in what case, despite what, what is contrasted with, before what, since when, what is similar, until when, instead of what, what is an alternative, except when, unless what.

Although many more archetypes could be devised (e.g., where to or who by), we believe that the list of questions we provided earlier is already rich enough to be generally representative, whereas more specific questions can always be framed by using the interrogative particles we considered (e.g., why, what). *Primary archetypes* can be used to represent any fact and abstract meaning (Bos, 2016). In contrast, the *secondary archetypes* can cover all the discourse relations between them (at least according to the PDTB theory).

### How to Measure the Degree of Explainability

Degree of Explainability (DoX) is a model-agnostic approach and metric, proposed by Sovrano and Vitali (2022c), to *objectively* evaluate explainability. It builds on *Achinstein’s theory of explanations* and the interpretation of illocution given by Sovrano and Vitali (2022b). DoX can quantify the degree of explainability of a corpus of texts by estimating how adequately that corpus could be used to answer in an illocutionary way an arbitrary set of archetypal questions about the explanandum.

In other words, Sovrano and Vitali (2022c) show how the degree of explainability of information depends on the number of *archetypal questions* to which it can adequately answer. In practice, DoX scores are computed by measuring and aggregating the *pertinence* with which a snippet of text can answer a (pre-defined) set of archetypal questions. Specifically, pertinence scores are obtained employing pre-trained *deep language models* for general-purpose answer retrieval (Karpukhin et al., 2020; Bowman et al., 2015) applied to a particular graph of triplets automatically extracted from text to facilitate this type of information retrieval.

### Explanatory Artificial Intelligence

An Explanatory AI (YAI) is an artificial intelligence program designed to generate user-centered, interactive explanations out of (possibly extensive) collections of explainable information (Sovrano & Vitali, 2022a). An example of YAI based on Achinstein’s theory of explanation is YAI4Hu^10^ (Sovrano & Vitali, 2021, 2022b).

YAI4Hu is a fully automatic explanatory tool that explains (pre-existing) documentation about an AI-based system. In particular, the textual content of such documentation is algorithmically reorganized and represented as a special hypergraph where information can be either explored through *overviewing* or searched via *open-ended questioning*. On the one hand, *open-ended questioning* can be performed by asking open-ended questions in English through a search box that uses the knowledge graph for efficient answer retrieval.

On the other hand, *overviewing* can be performed iteratively from an initial explanation by clicking on automatically annotated words for which more information is needed. In particular, annotated words are visible because they have a unique format that makes them easy to recognize. After clicking on an annotation, a modal window opens (see Fig. 1), showing a navigation bar of tabs containing explanatory overviews of the clicked annotated words. The information shown in the overview is as follows:

- A short description of the explained word (if available).
- The list of other words that are taxonomically connected.
- A list of pre-defined archetypal questions (e.g., why is this aspect/concept important, what is this aspect/concept, etc.) and their respective answers ordered by estimated pertinence (see section “Intelligent Explanation Generator: An Algorithm for the Identification of Pedagogically Useful Questions from Textbooks” for a definition of pertinence).

**Fig. 2:**
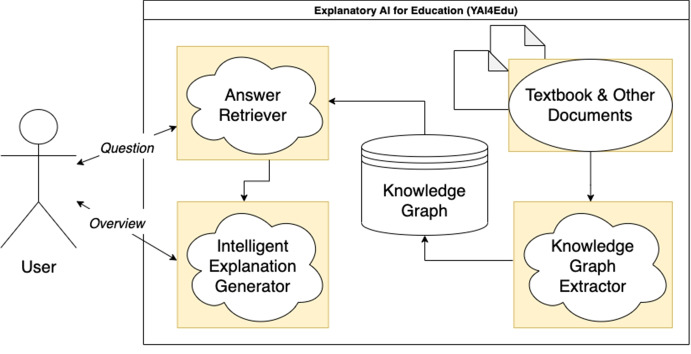
**Simplified Flow Diagram of YAI4Edu.** This diagram shows the main components of YAI4Edu. As in YAI4Hu, the user can ask questions and get overviews. However, differently, YAI4Edu uses a new component (i.e., the “Intelligent Explanation Generator”) for generating overviews, as described in section “Intelligent Explanation Generator: An Algorithm for the Identification of Pedagogically Useful Questions from Textbooks”

## YAI4Edu: a YAI for Improving the Explanatory Power of an Intelligent Textbook

The benefit of answering questions for learning has been shown in many studies (Rivers, 2021; Pan & Rickard, 2018), further supporting the assertion that explaining is akin to question-answering and that organizing contents on a question-answer base might be beneficial for the explainee. However, creating questions with proper detail that effectively helps students’ learning usually requires experience and extensive efforts (Shimmei & Matsuda, 2022).

For this reason, with the present work, we propose YAI4Edu (pipeline shown in Fig. 2), an extension of YAI4Hu (see section “Explanatory Artificial Intelligence”) to automatically transform static educative e-books (in PDF, XML or HTML format) into interactive intelligent textbooks by increasing their explanatory power. Similarly to YAI4Hu, YAI4Edu uses *open-ended questioning* and *overviewing* as main mechanisms for producing explanations. More specifically, interaction is given by: *i)* word glosses that can be clicked to open an *overview*; *ii)* a special kind of search box that allows the reader to get answers to any open-ended English question.

In particular, we exploit Achinstein’s theory of explanations to reorganize, on-demand, the contents of a textbook and connect it with external integrative resources (e.g., an encyclopedia) to provide the user with more useful explanations. We build a novel heuristic to identify and anticipate the most useful implicit questions an explainee might have about a textbook, organizing information accordingly. Indeed, according to theory (see section “Explanations According to Ordinary Language Philosophy”), what makes an Explanatory AI good at explaining is its ability to identify implicit and relevant questions to answer, i.e., its *illocutionary power*.

We developed an algorithm capable of automatically extracting questions from a textbook and identifying those that are neither too detailed (because they would otherwise only be answered in a minor part of the textbook) nor too general (because they would be answered inaccurately in the more detailed textual content). We did it by starting from the hypothesis that the most useful implicit questions a user may have about a collection of texts are those best answered by the whole collection.

**Fig. 3:**
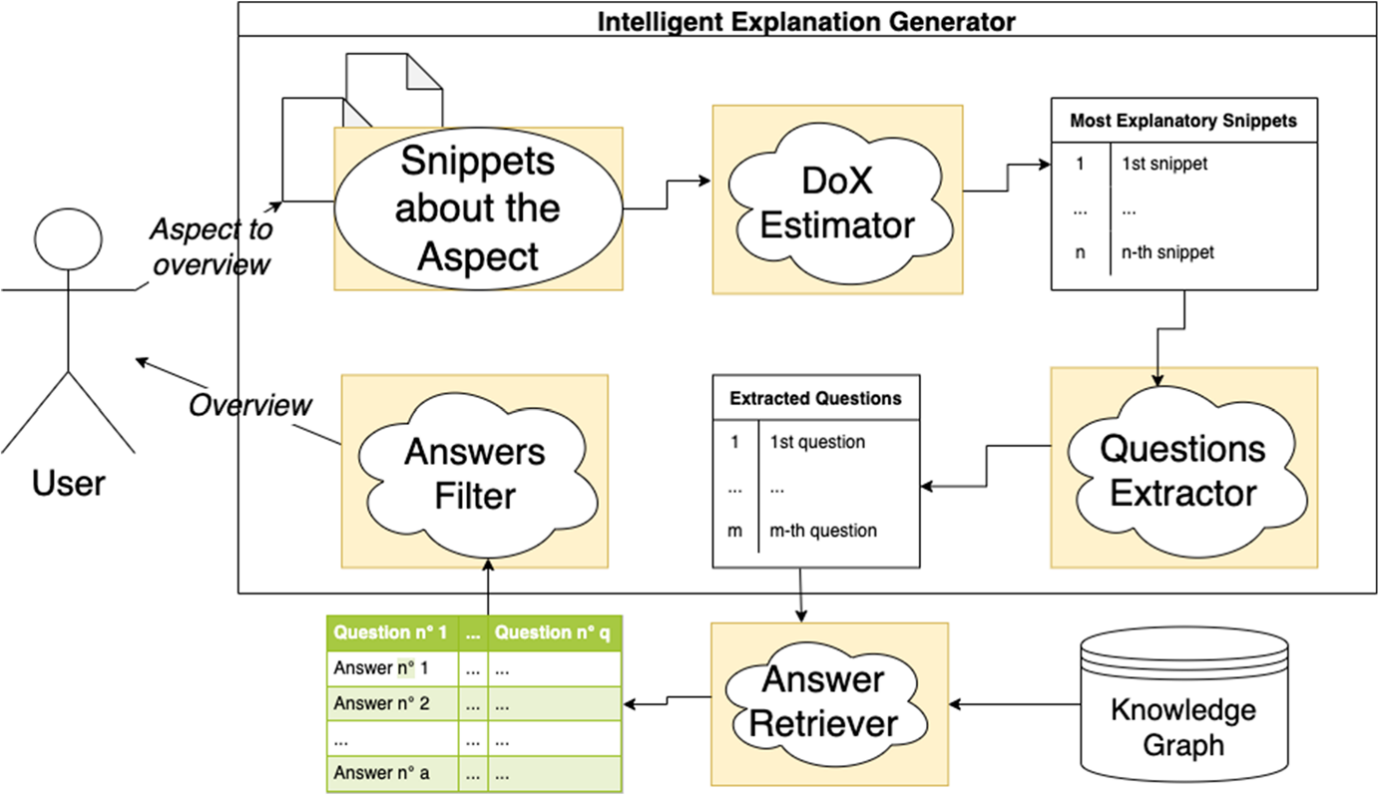
**Flow Diagram of the Intelligent Explanation Generator.** This diagram shows how an explainee can obtain an intelligent overview. First, the user decides which aspect of the explanandum to overview (i.e., by clicking on an annotated word available on the screen). Then the system extracts an explanation from the textbook or any other collection of texts (e.g., other textbooks, an encyclopedia) by using an AI for question extraction and an AI for answer retrieval as described in section “Answer Retrieval”

Suppose it is possible to quantify the explanatory power of a collection of explainable texts (i.e., through DoX; see section “How to Measure the Degree of Explainability”) and organize it accordingly by prioritizing the most explanatory contents (i.e., those with the highest DoX scores) over the others. In that case, it is also possible to identify which texts are the most explained within a corpus of documents and, therefore, the most useful questions to ask about the textbook. More specifically, for YAI4Edu, we developed several AI-based mechanisms that improve over the baseline YAI4Hu algorithm producing more pedagogically useful explanations via:

- intelligent overviewing (an example of overview is shown in Fig. 3), by automatically extracting the most useful questions that the textbook (or the support material) is answering;
- smart annotation generation, through the automatic identification of a glossary of words explained by the textbook’s contents.

In the following subsections, we will describe the technical details behind these new mechanisms and briefly summarize how the answer retrieval algorithm works. We also release the source code of YAI4Edu^11^ and the anonymized data collected to evaluate it (see section “Experiment”) under MIT license at https://github.com/Francesco-Sovrano/YAI4Edu.

### Automated Question Extraction for Intelligent Overviewing

According to theory, illocution in explaining involves informed and *pertinent* answering questions, implicitly relevant to the explanations and the explainee, understood as instances of archetypes. In section “Archetypal Questions in Linguistic Theories”, we presented different types of these archetypal questions identified by foundational linguistic theories.

Specifically, the secondary archetypes coming from discourse theory (Pyatkin et al., 2020) are meant to capture how Elementary Discourse Units (EDUs)^12^ are connected. In contrast, the primary archetypes coming from Abstract Meaning Representation theory^13^ (Michael et al., 2018) are meant to capture the informative components within the EDUs by possibly supporting answering basic questions such as “who did what to whom, when or where”. For example, from the sentence, “*The existence and validity of a contract, or any term of a contract, shall be determined by the law which would govern it under this regulation if the contract or term were valid,*” it is possible to extract the following discourse relation^14^ about contingency: *“In what case would the law govern under this regulation? If the contract or term were valid,”* and the following Abstract Meaning Representation (AMR) question-answer: *“By what is the existence and validity of a contract determined? By the law that would govern it under this regulation if the contract or clause were valid.”*

In particular, the discourse-based and AMR-based archetypal questions we need for YAI4Edu are extracted from sentences and paragraphs through a deep language model based on T5^15^ (Raffel et al., 2020), pre-trained on a multi-task mixture of unsupervised and supervised tasks.

Since vanilla T5 is not trained to extract discourse-based and AMR-based questions, we had to fine-tune T5 on some public datasets designed for this task. These datasets are QAMR (Michael et al., 2018) for extracting AMR-based questions and QADiscourse (Pyatkin et al., 2020) for discourse-based questions.

Most importantly, the QAMR and QADiscourse datasets are unrelated to any of the textbooks we will consider (see section “Case Study: A Textbook for Teaching How to Write Legal Memoranda”) for evaluating YAI4Edu. In particular, they do not contain any legal document or snippet of text written in legalese. In other words, we do not refine T5 on legal texts but instead train it on spoken English, given the high data availability. Therefore, training T5 the way we do does not imply any legal fine-tuning. Legal fine-tuning would require the costly extraction of a dataset of AMR- and discourse-based questions from legal texts, as well as ad-hoc adaptations of the theories of discourse and abstract meaning representation to legalese.

In particular, the QAMR dataset is made of 107,880 different questions (and answers), while the QADiscourse dataset is made of 16,613 different questions (and answers), as described in section “Archetypal Questions in Linguistic Theories”. The two considered datasets are tuples of $<s, q, a>$, where *s* is a source sentence, *q* is a question implicitly expressed in *s*, and *a* is an answer expressed in *s*. T5 is fine-tuned to tackle at once the following four tasks per dataset:

- extract *a* given *s* and *q*,
- extract *q* given *s* and *a*,
- extract all the possible *q* given *s*,
- extract all the possible *a* given *s*.

Specifically, we fine-tuned the T5 model on QAMR and QADiscourse for five epochs.^16^ The objective of the fine-tuning was to minimize a loss function measuring the difference between the expected output and the output given by T5. A mathematical definition of such a loss function is given by Raffel et al. (2020).

At the end of the training, the average loss was 0.4098, meaning that our fine-tuned T5 model cannot perfectly extract AMRs or EDUs from the text composing the training set. On the one hand, this is a good thing because it is likely that the model did not over-fit the training set. On the other hand, this clearly indicates that the questions extracted by our T5 model can be imperfect, containing errors that could propagate to the Intelligent Explanation Generator.

### Intelligent Explanation Generator: An Algorithm for the Identification of Pedagogically Useful Questions from Textbooks

Assuming that the goal of a textbook is to explain something to the reader, and based on the theoretical understandings expressed in section “Explanations According to Ordinary Language Philosophy”, our YAI4Edu is designed around the idea that organizing the explanatory space (i.e., the space of all possible bits of explanation) as clusters of archetypal questions and answers is beneficial for an explainee. In particular, YAI4Edu uses the following predefined interactions inherited from YAI4Hu to allow the user to explore this explanatory space:

- Open-ended Question-Answering: the user writes a question and receives one or more relevant answers.
- Aspect Overviewing: the user selects an aspect of the explanandum (i.e., contained in an answer) and receives as an explanation a set of *relevant* archetypal answers involving different aspects that can be explored as well. Archetypal answers can also be expanded, increasing the level of detail.

As hypothesized (cf. Hypothesis 1), archetypal questions that are too generic are unlikely to represent the explanatory goals of a sufficiently complex and elaborated collection of texts. The archetypal questions originally used by YAI4Hu for *overviewing* are too generic and predefined, frequently not adhering to the explanatory requirements of the overview. Therefore, considering the need for YAI4Edu to be pedagogically helpful, we designed a novel theoretically grounded AI algorithm to quantify how much an archetypal question is likely to represent the explanatory goals of a collection of texts. We called this algorithm the Intelligent Explanation Generator.

More specifically, instead of using predefined generic archetypal questions for our Intelligent Explanation Generator, we also consider more domain-specific ones automatically extracted from the knowledge graph through the AI for question-answer extraction. In particular, once an explanandum is chosen, the workflow of the Intelligent Explanation Generator consists of the following steps (also shown in Fig. 3).

- Step 1: the algorithm computes the DoX (see section “How to Measure the Degree of Explainability”) of all snippets of text about a given explanandum (i.e., an aspect to overview), finding the top *k* snippets with the highest DoX and also finding the archetypal questions extracted from them by the algorithm described in section “Automated Question Extraction for Intelligent Overviewing”;
- Step 2: for each question selected in the previous step, the algorithm identifies a set of pertinent answers within the text snippets. An answer is said to be pertinent to a question when its pertinence score^17^ is greater than a given pertinence threshold *p*;
- Step 3: the algorithm filters the pertinent answers, keeping the best *q* questions and *a* answers by executing the following sub-steps: *i)* questions that are too long are removed, i.e., questions whose length (without considering the length of the explanandum label) exceeds a threshold *L*; *ii)* if a question has some grammatical error, it is automatically corrected via Gramformer^18^, a deep neural network; *iii)* questions that are too similar are removed,^19^ prioritizing the questions extracted from the most explanatory snippets (i.e., those with the highest DoX) and the shortest questions; *iv)* answers that are too short or too long are removed; *v)* the questions with no valid answers are removed; *vi)* the answers that could be assigned to several questions are given to the question with the highest estimated pertinence; *vii)* for each question, only the *a* answers with the highest pertinence score are kept;*viii)* the questions are sorted by the decreasing pertinence of the first answer, and only the top *q* questions are kept.

Importantly, step 1 is performed before step 2 to reduce the asymptotic time complexity of step 2. Selecting the questions best answered by the corpus (step 2) has an asymptotic complexity $O(\vert Q_c \vert \cdot \vert S \vert )$ that grows with the number of questions extracted from the snippets of text, where *Q* is the set of questions about an aspect *c* to be explained and *S* is the set of snippets of text. Therefore, this complexity in the worst-case scenario (without step 1) can be quadratic in the size of the textbook or collection of texts, i.e., $O(\vert S \vert ^2)$.

Rather than having a quadratic complexity, a computationally simpler approach can perform an initial filtering procedure to consider only those questions coming from the paragraphs with the highest DoX (as step 1 does), thus converting $\vert Q_c \vert$ into a constant number independent from $\vert S \vert$. Hence, considering that computing the DoX of $\vert S \vert$ snippets of text has an asymptotic time complexity equal to $O(\vert S \vert )$, it follows that step 1 reduces the complexity of the Intelligent Explanation Generator to $O(\vert S \vert )$.

If Hypothesis 1 is true, then the Intelligent Explanation Generator will be able to produce better and more satisfying explanation overviews (than the baseline YAI4Hu). This is because it will be able to anticipate, in a sense, implicit questions the user may have.

Not all words, though, require an overview. That is because, in practice, only a tiny fraction of the words in a text are helpful to explain. Indeed, many words have common-sense meanings (e.g., the words: “and”, “first”, “figure”) and, therefore, should not be explained. Otherwise, the explainee might be overwhelmed by largely redundant and pointless information that would only hinder the usability of the YAI.

To intelligently avoid jotting down unnecessary words, our smart annotation mechanism only annotates those concepts and words that can be explained by (the knowledge graph extracted from) the textbook and other supplementary texts. More specifically, to understand whether a word should be annotated, the algorithm executes the following instructions:

- It checks whether the word is a stop word (i.e., a commonly used word such as “and” or “or”). If so, the word is not annotated.
- If the word is not a stop word, the algorithm generates its overview through the Intelligent Explanation Generator. Then, it computes the cumulative pertinence score of the answers composing the overview; if greater than a given threshold, it annotates the word.

This annotation mechanism is intended to remove noisy annotations and distractors so that the reader can focus only on the most central and well-explained concepts. Moreover, the cumulative pertinence score, used to understand whether a word should be annotated, can also be used to understand the topics in the corpus of documents that are better explained.

Consequently, the smart annotation mechanism and the Intelligent Explanation Generator are integral components of the YAI4Edu system. Notably, the Intelligent Explanation Generator tackles the challenge of selecting questions that balance between being too general or overly specific through the abovementioned filtering and refinement steps. By incorporating these steps, the Intelligent Explanation Generator prioritizes questions derived from the most informative excerpts, favors concise questions, and filters out excessively similar ones. This process aims to identify questions that align well with the explanatory objectives of the text collection, as discussed in section “Discussion: Results and Limitations”.

### Answer Retrieval

Similarly to YAI4Hu, YAI4Edu uses a pipeline of AI algorithms to extract a particular graph of knowledge from a collection of texts that an information retrieval system can exploit to answer a given question. In particular, a dependency parser detects all the possible grammatical clauses^20^ within the collection of texts. Each such clause stands for an edge of the knowledge graph.

In practice, these clauses are represented as combinations of subjects, templates, and objects/nominal modifiers.^21^ We refer to these as template-triplets. Specifically, templates consist of an ordered token sequence connecting subjects and objects/modifiers in a clause. In these templates, the subjects and the objects/modifiers are represented by the placeholders $``\{subj\}''$ and $``\{obj\}''$, respectively. The resulting template-triplets are a sort of function where the predicate is the body, and the object/modifier and the subject are the parameters.

Obtaining a natural language representation of these template-triplets involves replacing the instances of the parameters in the body. This natural language representation is then used as a possible answer for retrieval by measuring the similarity between its embedding and the embedding of a question. An example (taken from the case study discussed in section “Case Study: A Textbook for Teaching How to Write Legal Memoranda”) of template-triple is:

- Subject: *“A constitution”*
- Template: $``\{subj\}''$ specifically defines, empowers, and imposes $\{obj\}$ on each part of the governmental structure
- Object: *“limits”*

The resulting knowledge graph is imperfect because of the adopted extraction procedure. It may contain mistakes caused by wrongly identified grammatical dependencies or other issues. However, the likelihood of such errors occurring is relatively low. This is primarily attributed to the high accuracy of the model we used for part-of-speech tagging.^22^

To increase the interoperability of the extracted knowledge graph with external resources, we formatted it as an RDF^23^ graph. In particular, RDF has features that facilitate data merging even if the underlying schemas differ. To format a graph of template triplets in an RDF graph, we performed the following steps:

- We assigned a URI^24^ to every node (i.e., subject and object) and edge (i.e., template) of the graph by lemmatizing the associated text. We assigned an RDFS^25^ label to each URI corresponding to the associated text.
- We added special triplets to keep track of the sources from which the template-triplets were extracted so that each node and edge can refer to its source document or paragraph.
- We added sub-class relations between composite concepts (syntagms) and the simplest concepts (if any) composing the syntagm. For instance, “*contractual obligation*” is a sub-class of “*obligation*”. Therefore, we employ the RDFS predicate $``\{subj\}rdfs:subClassOf ~\{obj\}''$ as a template for the triplet that signifies this sub-class relationship.

For more technical details about how we performed all the steps mentioned above to convert the template-triplets into an RDF graph, please refer to Sovrano et al. (2020) or the source code of YAI4Edu.

Finally, the algorithm to retrieve answers from the extracted knowledge graph is based on the following steps. Let *C* be the set of concepts in a question *q*, $m=<s,t,o>$ be a template-triplet, $u=t(s,o)$ be the natural language representation of *m*, also called an *information unit*, and *z* be its source paragraph. DiscoLQA retrieves answers by finding the most similar concepts to *C* within the knowledge graph, retrieving all their related template-triplets *m* (including those of the sub-classes), and selecting, among the natural language representations *u* of the retrieved template-triplets, those that are likely to answer *q*. The likelihood of *u* answering *q* is estimated by computing the cosine similarity between its contextualized embedding $<u,z>$ and the embedding of *q*. If $<u,z>$ is similar enough to *q*, then *z* is said to answer *q*.

The embeddings of $<u,z>$ and *q* are obtained through a deep language model specialized in answer retrieval pre-trained in ordinary English to associate similar vectorial representations to a question and its correct answers. The pre-trained deep language model we employed in implementing YAI4Edu is a variation of MiniLM (Wang et al., 2021) published by Reimers and Gurevych (2019).

## Case Study: A Textbook for Teaching How to Write Legal Memoranda

To showcase and evaluate YAI4Edu, we considered a case study in the intersection between AI and law. In particular, we applied YAI4Edu to the following material explaining, among other things, how to write a legal memorandum in a U.S. legal context for a veteran’s PTSD disability claim:

- 22 pages excerpted from the textbook “*United States Legal Language and Culture: An Introduction to the U.S. Common Law System*” (Brostoff & Sinsheimer, 2013 pp. 47-60, 93-96, 101-103, 131-132).^26^
- 5,407 open access web pages about concepts related to the U.S. legal system coming from the encyclopedia of the *Legal Information Institute of the Cornell Law School*^27^ (5,406 web pages) and Wikipedia^28^ (1 web page).
- 11,198 legal cases on PTSD disability claims taken from the official website of the Board of Veterans’ Appeals (BVA).^29^

Altogether, the included material, comprising more than 16,000 documents, complements the primary teaching material on which YAI4Edu focuses, i.e., the excerpts of the selected textbook. In particular, the textbook is used in “Applied Legal Analytics and AI,”^30^ an interdisciplinary course at the University of Pittsburgh, co-taught by instructors from the University of Pittsburgh School of Law and Carnegie Mellon University’s Language Technologies Institute. It provides “a hands-on practical introduction to the fields of artificial intelligence, machine learning, and natural language processing as they are being applied to support the work of legal professionals, researchers, and administrators.”

Teaching how to write a legal memorandum for the U.S. legal system is a course objective, in part, because in a *common law* system, such as the American one, the use of AI assists practitioners in efficiently retrieving legal cases for constructing arguments (Phelps & Ashley, 2022). A *legal memorandum* is an organized document that summarizes relevant laws to support a conclusion on a particular legal issue. Writing it can require legal practitioners to navigate through large databases of cases, i.e., to retrieve the definitions of technical and specific concepts or to understand which argumentation patterns are most common in a particular context. Indeed, some of the distinguishing features of legal writing are:

- Authority: The writer must back up assertions and statements with citations of authority (i.e., precedents and other decided cases).
- Argument re-use: A more effective memorandum may reuse existing documents as templates or argumentation patterns.
- Formality: The written legal document should be properly formatted according to existing standards.

Hence, legal practitioners may now be required to learn how to efficiently and effectively interact with existing AI-based technological solutions for information retrieval to speed up legal writing and to learn the complexities of legal writing. Given the task’s complexity and the course’s goals, we envisaged that it might be of utmost relevance and utility to design and create a tool such as YAI4Edu that could ease the acquisition of the necessary knowledge for a student to learn legal writing.

Specifically, YAI4Edu should help students understand, from real examples, how to write a legal memorandum comprising legal arguments to defend or attack a claim. In particular, students have to understand how to use statutes, read and summarize cases, synthesize cases, draft a legal memorandum, and use legal concepts in writing. This may involve learning legal concepts as well as skills of making arguments with concrete cases selected using legal information retrieval tools, as is typical in the U.S. legal context.

**Table 1:** **Useful statistics about the case study**: the column “Documents” shows the number of documents

	Documents	Extracted Questions	Concepts	YAI Concepts	KG Size	Tokens	Tokens per Doc
Textbook & Web Pages	5,408	246,747	115,110	3,407	2,059,145	707,317	130.79
+ Legal Cases	11,198	1,062,716	1,410,694	4,579	52,987,778	28,630,575	2,556.75
= Total	16,606	1,309,463	1,525,804	7,986	55,046,923	29,337,892	1,766.7

“Extracted Questions” provides the number of different questions extracted by the algorithm described in section “Automated Question Extraction for Intelligent Overviewing”. “Concepts” shows the number of different concepts/topics identified in the collection of documents. “YAI Concepts” provides the number of topics that can be explained by the algorithm described in section “Intelligent Explanation Generator: An Algorithm for the Identification of Pedagogically Useful Questions from Textbooks”. “KG Size” shows the number of RDF triplets composing the knowledge graph (KG) extracted by the algorithm described in section “Answer Retrieval”. “Tokens” shows the total number of tokens (e.g., words) in the collection of documents. “Tokens per Doc” shows the mean number of tokens per document

In particular, applying YAI4Edu to the collection of documents mentioned above, we extracted a knowledge graph of 52,987,778 RDF triplets from the BVA cases and a knowledge graph of 2,059,145 RDF triplets from the textbook excerpts and the other web pages; other statistics are shown in Table 1.

Thanks to the *compositional* nature of RDF graphs, we can combine smaller individual graphs into larger ones without altering the original meaning or semantics. Taking advantage of this feature, we enriched the graph with about ten manually added RDF triplets to fill identified knowledge gaps, such as “memo” being a synonym for “memorandum”. These gaps surfaced during software testing.

However, we have not thoroughly analyzed the whole extracted RDF graph. As a result, other knowledge gaps may yet emerge. Despite our incomplete analysis, we believe this property of *compositionality* of the knowledge graph used by YAI4Edu is of utmost importance. Indeed, it enables manually correcting any error produced during the graph extraction and easily integrating it with additional or missing knowledge.

**Fig. 4:**
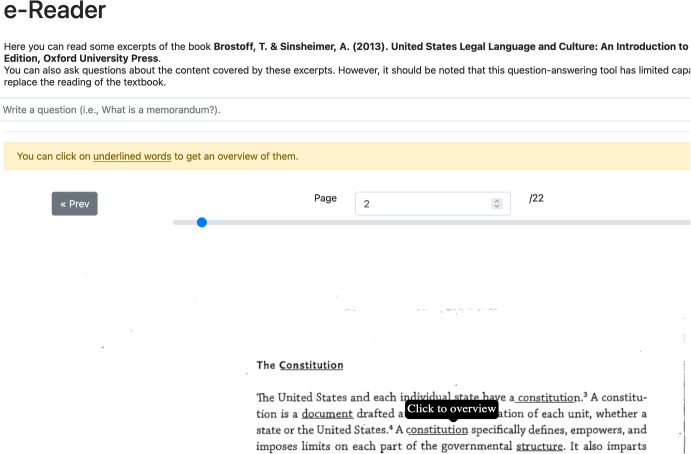
**Landing page of YAI4Edu applied to the case study.** This figure contains a screenshot of the annotated textbook (Brostoff & Sinsheimer, 2013) and the input for *open-ended questioning*. Clicking on underlined words opens an explanatory *overview*, an example of which is shown in Fig. 1

This knowledge graph helped to build an interactive and intelligent version of the textbook, as described in section “YAI4Edu: a YAI for Improving the Explanatory Power of an Intelligent Textbook” and shown in Fig. 2. There, an input box for *open-ended questioning* and annotated (i.e., underlined) words for *overviewing* (shown in Figs. 4 and 1) provide the user with interactive elements to obtain intelligent explanations without breaking the structure of the textbook.

The choice of hyper-parameters (i.e., the pertinence threshold *p* or the question similarity threshold *s*; cf. section “Intelligent Explanation Generator: An Algorithm for the Identification of Pedagogically Useful Questions from Textbooks”) of the Intelligent Explanation Generator is focused on generating concise and compact explanations. In particular, the hyper-parameters chosen for this instance of YAI4Edu are the following: *i)* number of snippets with the highest DoX to consider $k=10$; *ii)* answer pertinence threshold $p=.57$; *iii)* maximum overview question length $L=50$;*iv)* question similarity threshold $s=.95$; *v)* minimum and maximum answer length equal to 150 and 1000; *vi)* maximum number of questions per overview $q=4$; *vii)* maximum number of answers per overview question $a=2$.

**Fig. 5:**
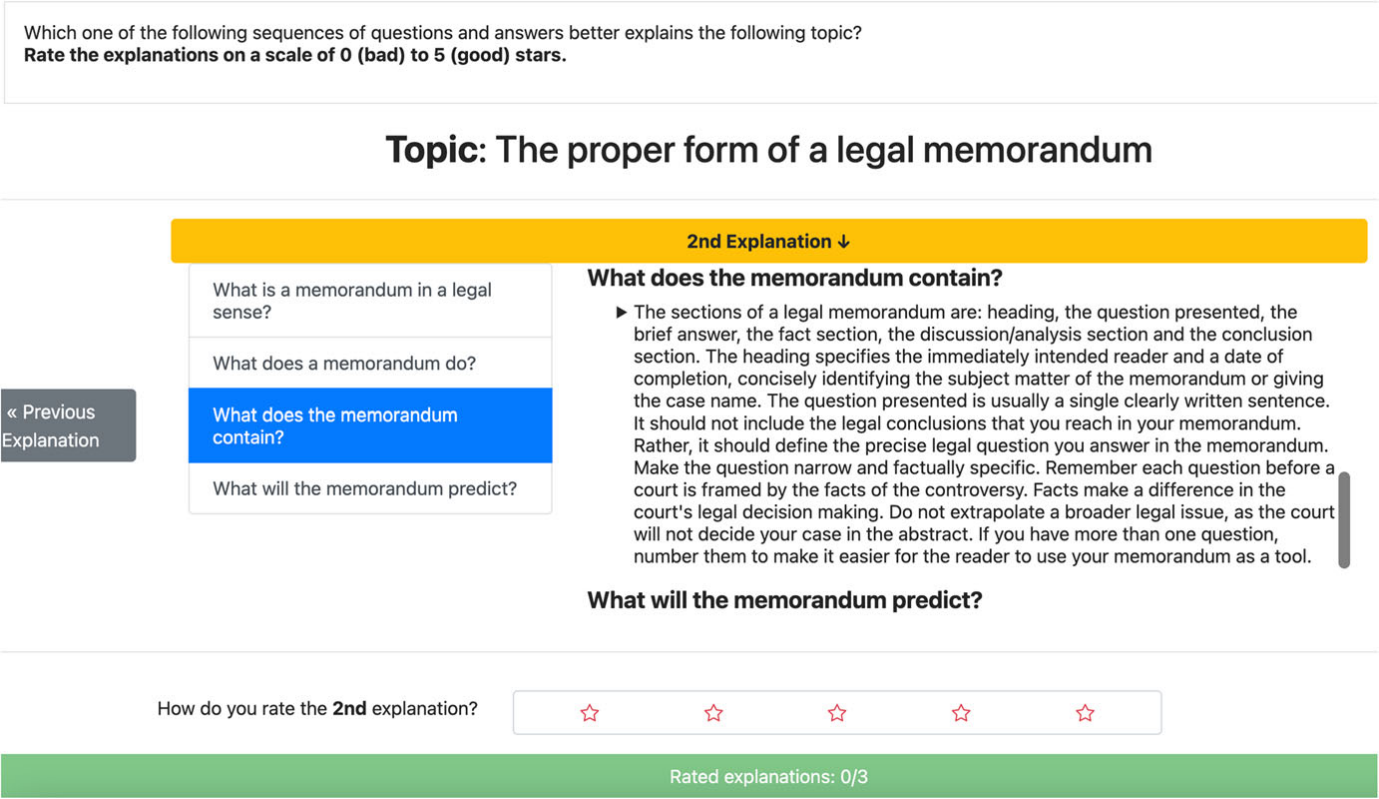
**Screenshot of the web application used during the experiment.** This figure shows what the participants in the user study see during the experiment

## Experiment

Explanations and explanatory tools may be complex artifacts whose quality depends on a wide range of different factors (Sovrano et al., 2020), including:

- the quality of the explainable information;
- the logic used for presenting this information, namely the set of rules employed to present the information in a meaningful and coherent manner, which we refer to as the *presentation logic*;
- the quality of the interface.

In particular, with this experiment, we are interested in evaluating the presentation logic used by YAI4Edu for selecting and reorganizing questions and answers into explanations.

Sovrano and Vitali (2022a) have already demonstrated, with several examples and experiments, a user-centered YAI is better than one-size-fits-all and static explanations. In this experiment, instead of evaluating the whole interactive e-book with a rather time-consuming test, we focused on evaluating the one feature of YAI4Edu that should be responsible for improving the explanatory power of the e-book: the Intelligent Explanation Generator. According to theory (see section “Explanations According to Ordinary Language Philosophy”), what makes a YAI good at explaining is its ability to identify implicit and relevant questions to answer, i.e., its illocutionary power.

Therefore, in this experiment (which is a *within-subjects* user study), we directly ask real students to rate explanations (on a scale of 0 to 5 stars) for how well they adequately explain a given topic, as shown in Fig. 5. We do it to understand the extent to which the explanations generated by our Intelligent Explanation Generator are satisfactory and whether they are better than baseline explanatory strategies, as stated by Hypothesis 1. More details about the experiment can be found in Appendix A.

The two baselines against which the Intelligent Explanation Generator is compared are variants of the same. They use the same sequence of steps to generate their explanations, apart from the step responsible for selecting the explanatory questions, which is different. These two baselines are:

- An explainer that uses randomly chosen questions to organize the contents of an *overview*. This explainer randomly selects $q=4$ questions, setting the maximum question length to $L=\infty$ and using a lower answer pertinence threshold $p=.3$ (and not $p=.57$ as in the Intelligent Explanation Generator). This prevents the number of questions from diminishing too much due to not finding sufficiently relevant answers.
- YAI4Hu’s generic overview generator, which instead employs pre-defined and very generic archetypal questions, using the same four questions (i.e., what is it, how is it, where is it and why) for each topic.

Given the case study at hand (see section “Case Study: A Textbook for Teaching How to Write Legal Memoranda”), the main objective of the explanatory contents is to explain how to write a legal memorandum appropriate for the U.S. legal system and a veteran’s PTSD disability claim. The excerpts of the considered textbook are about legal writing, while the collection of legal cases of the BVA are about PTSD disability claims. Thus, we can say that some of the goals of the YAI for this case study are to explain: *i)* what is the proper form of a legal memorandum; *ii)* what sections should be included in a legal memorandum; *iii)* what legal standard does a veteran need to satisfy for a disability claim; *iv)* what are the elements of the legal standard a veteran needs to satisfy for a disability claim; *v)* what issues do the required elements of a disability claim raise; and *vi)* what kinds of legal arguments are appropriate for resolving such issues.

Considering that we need an experiment lasting a maximum of 10 minutes (in order to minimize costs: each participant cannot be paid less than 6$\pounds$ per hour on Prolific), we chose the following three topics for evaluating the explainers:

- Topic 1: The proper form of a legal memorandum.
- Topic 2: The effects of a disability.
- Topic 3: The elements of the legal standard a veteran needs to satisfy for a PTSD disability claim.

In particular, the explanations for the first two topics are extracted from the textbook and web pages (the first is better explained by the textbook, the second by the web pages). In contrast, the explanations for the third topic are extracted from legal cases. For more details about the explanations used in the experiment, see Table 2.

**Table 2:** **Questions used during the experiment.** This table shows the questions extracted from the three explainers of the experiment. Specifically, “intelligent” stands for the Intelligent Explanation Generator, “generic” is the YA4Hu explainer, and “random” is the explainer that uses random questions. Note that an explainer uses fewer than four questions (the maximum) for its explanations whenever it does not find four questions with relevant answers in the knowledge graph. The sum of the relevance scores of all answers that make up each explanation is reported in the column “Cumulative Relevance”

Topic	Explainer	Cumulative Pertinence	Questions
The proper form of a legal memorandum	generic	2,81	What
			How
			Why
	random	3,79	What is the result of a memorandum to a partner in the same firm?
			In what manner is a memorandum of points and authority usually mandatory?
			What does the memorandum usually include?
	intelligent	4,61	What is a memorandum in a legal sense?
			What does a memorandum do?
			What does the memorandum contain?
			What will the memorandum predict?
The effects of a disability	generic	3,47	What
			How
			Why
	random	4,92	What is the reason schools must determine if they have a covered disability under the Act and if that disability is severe enough?
			What can a partial disability be?
			What is an example of state statutes relating to disability retirement?
			In what manner can a partial disability be permanent?
	intelligent	5,65	What is Disability Law?
			What is disability?
			What is the result of disability in a legal sense?
			What does the disability prevent?
The elements of the legal standard a veteran needs to satisfy for a PTSD disability claim	generic	4,58	What
			How

Topic	Explainer	Cumulative Pertinence	Questions
			Why
			Where
	random	4,09	Why is element two met?
			Who is the first element of a service connection?
			Who found that the Veteran has met the first two elements of service connection?
	intelligent	5,21	What is the first element of a service connection?
			What are the elements of service connection?
			What are elements of the legal standard a veteran needs to satisfy for a PTSD disability claim?
			What is an element of appeal?

## Discussion: Results and Limitations

Through Prolific, we gathered 130 participants, all students aged between 19 and 38. 28 participants were discarded for the reasons stated in Appendix A. Eventually, 102 valid submissions were collected.

**Fig. 6:**
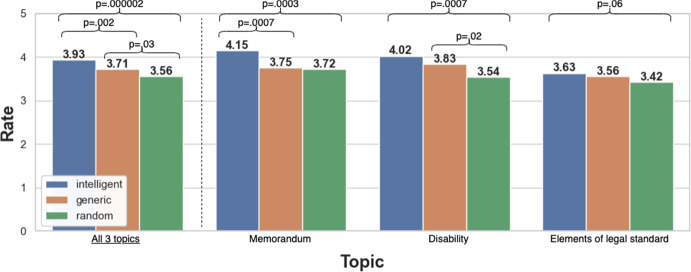
**Experiment results.** This figure contains bar charts showing the average scores and the statistically significant *P* values (indicated above the curly brackets) for each topic and explainer. For a list of all *P* values, see Table 3. On the left side of the figure, one can see the results obtained by aggregating the scores for all three topics. In particular, the bar plot labeled as “memorandum” refers to the first topic, “disability” to the second topic, and “elements of legal standard” to the third topic

The results (shown in Fig. 6) indicate that the *intelligent* explainer received the highest rates, followed by the *generic* one; the worst was the *random* explainer. To further validate the results and verify that the improvements of the *intelligent* explainer over the baselines are statistically significant, we performed one-sided Mann-Whitney U-tests (a non-parametric version of the t-test for independent samples) whose results are summarized in Fig. 6. Results clearly show that, assuming $P < .05$ is enough to assert statistical significance, the *intelligent* explainer is superior to the baselines regarding perceived explanatory power in all three chosen topics. For more details on statistical tests, see Appendix B.

Interestingly, looking at the topics separately, we also have statistical evidence showing that the *intelligent* explainer is better than the *generic* and the *random* explainer for the first two topics, but not enough statistical evidence for the last one. This may be because the variance of the cumulative pertinence of the explanations about the third topic (see Table 2) is too low. Alternatively (and more likely), this may also be because the explanations about the last topic were extracted from a corpus of legal cases rather than textbooks or other educational contents as the other two, thus being harder to explain. In particular, this intuition is corroborated by the statistics of Table 1, where one can see that the legal cases have a ratio of explained concepts close to 0.32%. In contrast, the textbook and web pages have 2.96% (10 times greater). This difference in explainability between the two documents corpora may impact the quality of extracted explanations. Indeed, as pointed out by some qualitative feedback, the explanations extracted from the BVA cases contain too much (unexplained) technical jargon and too long sentences (e.g., “the first topic was easy to understand also the second one, the problem with the last one it was too long and was not straight to the point.”).

This shows that the most useful implicit questions a user may have about a collection of texts will likely be those best answered by the whole collection. Furthermore, even if the *random* explanations have a cumulative pertinence score greater than *generic* explanations (at least for the first two topics, as shown in Table 2), they are evaluated as worse explanations nonetheless. This evidence further supports Hypothesis 1, showing that too specific archetypal questions may be less effective than generic ones at explaining and that intelligently balancing between generality and specificity is needed, as also suggested by some qualitative feedback:

- “All of the [random] explanations were OK, but improvement is needed. They lack a sense of direction. It’s like they go around mountains to prove one single point. All [generic] explanations were easy to decode and were straight to the point. All [intelligent] explanations were a mixture of first and second explanations.”
- “The [random explanations] proved unsatisfactory for all three topics: the explanations do not follow a logical order, are incomplete, and often contain incorrect or irrelevant elements. The [generic and intelligent explanations] are quite complete. The [generic explanations] seem to fit more practical questions, while [intelligent explanations] fit more theoretical ones. I believe [intelligent explanations] are preferable for the topic at hand.”

## Limitations and Future Work

One limitation of our study pertains to its scope, encompassing both the duration of the study and the number of participants involved. Specifically, the study was conducted over a brief 10-minute period and comprised a relatively small group of 102 individuals. This restricted scope raises concerns regarding the generalizability of our findings. Furthermore, our research primarily concentrated on participant ratings and qualitative feedback, without directly measuring changes in learning outcomes.

As a result, while our results offer initial insights into the potential applications of educational tools like YAI4Edu, they should be approached with caution. In order to validate and expand upon these preliminary findings, it is necessary to conduct more comprehensive studies over longer time-frames, involving larger and more diverse participant groups. However, previous research (Sovrano and Vitali, 2022a) provides compelling evidence supporting the notion that user-centered illocution significantly enhances the effectiveness of YAI software, leading to more efficient explanations and improved learning outcomes, as suggested also by qualitative feedback.

It is worth noting that even though qualitative feedback was optional, a notable 81% of users provided feedback. Interestingly, 19 participants left highly positive feedback (e.g., “the explanations were superb and of good quality”) without suggesting any improvement or explaining their rates, unlike the remaining 64 users. Overall, we identified **6 major suggestions for improvement**:

- **Avoid long and redundant explanations**: suggested by 32 participants;
- **Avoid or explain legal jargon**: 24;
- **Avoid generic or incomplete information**: 18;
- **Use simpler questions**: 9;
- **Provide examples when explaining**: 7;
- **Provide better organized and compartmentalized contents**: 5.

The complaints were primarily about too-long explanations, unexplained legal jargon, or generic/incomplete information. Some qualitative feedback comments ask for more conciseness, and others for less. Some participants preferred *generic* explanations over *intelligent* ones. Interestingly, one could turn this into a feature if the system could offer users a choice of generic or intelligent explanations.

The qualitative feedback was extremely helpful in identifying the main problems and limitations of YAI4Edu and the baselines, pointing to future work. In particular, we believe YAI4Edu’s *smart annotation* mechanism can partially mitigate the jargon problem by explaining it. However, we could not verify this with the experiment because it was set to take 10 minutes, so intelligent annotations were excluded. See Appendix A for more details on qualitative feedback.

Feedback has also indicated that YAI4Edu could significantly improve in terms of efficiently integrating retrieved information into a coherent structure. As it stands, explanations often tend to be lengthy, repetitive, and disjointed. The introduction of generative AI like ChatGPT might serve as a valuable solution to these issues, re-elaborating the information retrieved by YAI4Edu. Indeed, ChatGPT has the potential to produce content that is not only concise and coherent but also well-organized. It can adeptly avoid or interpret complex (legal) jargon, presenting content in a well-structured and compartmentalized manner. These features could address at least three out of the six primary improvement areas identified in the qualitative feedback. As a result, the integration of such AI technology could significantly enhance the effectiveness of YAI4Edu, as shown in Sovrano et al. (2023).

It should be noted, however, that while ChatGPT can complement YAI4Edu, it cannot fully replace it. There are unique challenges associated with using large language models like ChatGPT to generate explanations from input documents, such as textbooks. These challenges include memory limitations and the risk of generating inaccurate or copyrighted content. Unlike ChatGPT, YAI4Edu primarily relies on information retrieval technologies, eliminating the need to input the entire context of the explanation into the language model. This is particularly important also for standard-sized textbooks, where memory constraints can easily become an issue, as discussed in the study by Sovrano et al. (2023).

Future studies also need to focus on several factors to secure more robust results. First, we need further investigation into the effectiveness of our random explanations generator as a baseline. Given the feedback regarding redundant explanations and the need for better-organized content, exploring sophisticated alternatives like ChatGPT might provide a stronger baseline for comparison with YAI4Edu.

Second, our research did not integrate commonsense and domain-general knowledge into the YAI pipeline, making the system highly dependent on well-written, logically coherent texts. This dependency sets a high standard for the source text, potentially limiting the extensibility of our work. Addressing this concern might be possible with the help of large language models, such as ChatGPT, which could potentially excel in tasks similar to YAI4Edu. This holds particularly true for unstructured texts and those not professionally written, offering promising avenues for future research.

## Conclusion

In this paper, we presented YAI4Edu, an Explanatory AI for the automatic generation of interactive and adaptive e-books for education. To create YAI4Edu, we enhanced the YAI proposed by Sovrano and Vitali (2022b), making it more explanatory and more intelligent through a novel AI, called Intelligent Explanation Generator, for extracting explanations that are neither too specific nor too generic.

The idea behind our work is that explaining is akin to question answering, so the more questions an intelligent textbook can answer, the more it can explain. In other words, the explanatory power of a book can be improved by reorganizing it interactively, firstly to help readers identify the most critical questions to be answered about the textbook, but also to get answers to their questions. In particular, YAI4Edu uses the Intelligent Explanation Generator, an efficient pipeline for automatic (archetypal) question extraction and answer retrieval, to select these important questions and use them to reorganize the contents of a textbook. This is done following Hypothesis 1 for which the most useful questions a user may have about a collection of texts (e.g., a textbook) are best answered in the collection as a whole.

We applied YAI4Edu to a textbook for teaching how to write legal memoranda appropriate for the U.S. legal system and a PTSD disability claim. This was integrated with 5,406 external web pages from a legal encyclopedia and 11,198 legal cases from the Board of Veterans’ Appeals. Then, to show that our novel strategy for generating explanations is more effective than baseline approaches, we devised a within-subjects user study involving more than 100 English-speaking students who evaluated explanations about three different and relevant topics. The final results were in many cases statistically significant and always in favor of our Intelligent Explanation Generator, suggesting that Hypothesis 1 is true.

The impact of our YAI software pipeline extends to various sectors, including education, professional training, and industry. Within the education sector, our tool enhances the use of textbooks by seamlessly integrating external content, facilitating efficient exploration and retrieval of information through answer retrieval mechanisms. In professional training, it has the potential to elucidate complex concepts and procedures, thereby expediting knowledge acquisition, particularly in highly technical domains. In the industrial context, our tool can simplify technical documentation and improve customer support, as suggested by Sovrano and Vitali (2022b); Sovrano et al. (2023).

Looking ahead, we foresee a notable shift in education with interactive e-books and AI-generated content supplementing or replacing traditional textbooks. These tools promise personalized, up-to-date learning experiences. Rather than replacing educators, these AI tools could augment their capabilities by providing personalized instruction at scale. They could explain basic concepts and answer common questions, thus allowing educators to focus on more complex topics, critical thinking, creativity, and personalized guidance.

## Appendix A Experiment Details

The experiment consisted of a 10-minute *within-subjects* user study where the explanations generated by two baseline explainers and YAI4Edu were evaluated by English-speaking students assembled with Prolific^31^, an online platform that helps recruit paid participants for online research.

Through Prolific, we gathered 130 participants, all students aged between 19 and 38, each paid $\pounds$1. 28 of the participants were discarded for the following reasons:

- 26 participants were discarded because they were too quick (i.e., they spent less than 4 minutes completing the evaluation of all topics) or they skipped at least one topic (i.e., they spent less than 35 seconds on it without being a legal expert);
- 2 participants were rejected because they reported poor knowledge of written English (i.e., A1 or A2) or wrote non-grammatical qualitative feedback.

Eventually, 102 valid submissions were collected.

Of these 102 participants:

- 46 were males, 52 females, 1 identified itself as other/non-binary, and three preferred not to say;
- 81% stated that their written English proficiency level is C1 or C2, while 16% indicated a B2 level;
- 86% rated their knowledge of the U.S. legal system with a score lower than or equal to 3;
- 86 said they are not legal experts;
- 68 reported that they have low or no experience with legal writing, 29 wrote they have some experience, and five that their experience with legal writing is high.

Participants were required to be fluent in English, be resident in English-speaking countries (i.e., USA, UK, Ireland, Australia, Canada, New Zealand, South Africa), use a device with a large screen (e.g., a laptop, a desktop computer, a tablet in landscape mode), be at least 18 years old, and possess a student status and a minimum approval rating of 75% on Prolific.

Moreover, at the beginning of the study, they were asked to provide the following information anonymously: age; gender; whether they are legal experts; their proficiency in written English, on a scale of A1 (very low) to C2 (very high); their experience in legal writing (from *none* to *high*); how they would rate their knowledge of the U.S. legal system on a scale of 0 (bad) to 5 (good) stars.

It is important to note that during the experiment, for each topic, each participant could compare the explanations generated by all three explainers, provided in random order to avoid unwanted biases that might be caused by some explanations being shown before or after others. Furthermore, using a predefined random seed, the explanations generated by the random explainer were the same for all participants.

In the experiment, participants were not informed about the origin of the explanations they evaluated, specifically whether they came from random, generic, or intelligent generators, nor were they made aware of the existence of these three different generators. Each explanation was to be evaluated on a 0 to 5-star scale, with 0 representing “bad” and 5 indicating “good”, as depicted in Fig. 5. Once the testing was completed, participants were also invited to share their qualitative feedback in an open-ended format.

Some of the most interesting feedback examples are the following:

- “The more lengthy explanations offer more details and give the reader a greater understanding but can feel a bit harder to read rather than the [generic explanations].”
- “Dividing the topic into sections is good if the [questions] are relevant and make sense. Relatively simple explanations supported by evidence are better, in my opinion.”
- “The longer explanations were more detailed and more understandable. The shorter definitions were also understandable and compact, but the law should be detailed. ”
- “I particularly liked the layout that included ’what, why, how’, as it made the explanations easy to follow. [...] The headings with long sentences lost me before I began, and I found it hard to decipher the explanations.”
- “I would make [explanations] shorter.”
- “The answers should be less vague and focus more on details.”
- “The explanations with very long or involved subheadings were difficult to follow. Often, when there are large blocks of text, my mind tends to get overwhelmed - simply making shorter subheadings and adding more paragraph breaks to the explanations helps with this.”
- “The answers to the questions could have been more concise, for instance, in the first topic on what a memorandum is, though I found [intelligent] and [generic explanations] to be similar in their verbiage. I found [generic explanations] always easier to understand across the board. Because of its simplistic presentation, I did not spend time focusing on unnecessary details”
- “The explanations are a bit difficult to follow as they are long, so as a reader, you get lost in the middle of the explanation and forget what you just read on top.”
- “They were pretty straightforward and easy to understand, especially because descriptions relating to the law or topics that are difficult to understand are always filled with difficult jargon, but this simplified version made it easy to understand.”
- “Generally, the explanations were full but a bit difficult to digest. There were cases in which not enough was explained to fully understand what a specific legal term meant and encompassed. The explanations that had a short and quick explanation of some legal terms followed by a more prolonged and detailed explanation were, for me, the easiest to grasp.”

## Appendix B Statistical Tests

Complementing Fig. 6, Table 3 provides the extended results of Mann-Whitney U-tests conducted on three different comparisons: Random vs Generic, Random vs Intelligent, and Generic vs Intelligent. We carried out these tests both on the aggregate data across all three topics (memorandum, disability, elements of legal standard), and on each topic individually. For each comparison, the results include the U statistic, the p-value, the Common Language Effect Size (CLES), and the Rank-biserial Correlation.

**Table 3:** **Effect Size of Statistical Tests.** This table includes the comparison groups, the tested hypothesis, the U statistic, p-value, CLES, and Rank-biserial Correlation for each test

Topic	Comparison	Alternative Hypothesis	U	p-value	CLES	Rank-biserial Correlation
All	Random vs Generic	Random is less	43039.0	0.036	0.46	0.08
	Random vs Intelligent	Random is less	37340.5	$<0.001$	0.40	0.2
	Generic vs Intelligent	Generic is less	41019.5	0.0027	0.44	0.12
Memorandum	Random vs Generic	Random is less	5192.5	0.491	0.50	0.002
	Random vs Intelligent	Random is less	3838.0	$<0.001$	0.37	0.26
	Generic vs Intelligent	Generic is less	3925.5	0.0007	0.38	0.25
Disability	Random vs Generic	Random is less	4416.5	0.026	0.42	0.15
	Random vs Intelligent	Random is less	3930.0	0.0008	0.38	0.24
	Generic vs Intelligent	Generic is less	4728.5	0.118	0.45	0.09
Elements of	Random vs Generic	Random is less	4767.0	0.142	0.46	0.08
Legal Standard	Random vs Intelligent	Random is less	4597.5	0.068	0.44	0.12
	Generic vs Intelligent	Generic is less	5021.0	0.327	0.48	0.03

The interpretation of the results across all three topics reveals a consistent pattern. On the whole, we find a significant difference favoring the Generic over the Random group, and the Intelligent group over the Random group. The effect sizes of these differences range from small to medium, as indicated by the Common Language Effect Size and Rank-biserial Correlation. The differences between the Generic and Intelligent groups are also significant, albeit smaller.

When it comes to the specific topic of “Memorandum”, there is no notable difference between the Random and Generic groups. However, both the Random and Generic groups score significantly lower than the Intelligent group, with small to medium effect sizes.

In the case of “Disability”, the Random group performs significantly worse than both the Generic and Intelligent groups, with small to medium effect sizes. However, the Generic and Intelligent groups do not significantly differ.

For the “Elements of legal standard” topic, no significant difference is found between the groups, but the Random group does trend lower than the Generic and Intelligent groups, with small effect sizes. The Generic and Intelligent groups are also very similar in performance.

In all, while we see some variation by topic, the overarching pattern is that the Intelligent group consistently outperforms the Random group, and the Generic group falls somewhere in between.

Since we are performing multiple comparisons with Mann-Whitney U-tests, the chances of a false comparison increase. Some statistical tools that are used in this case to reduce the chance of a type I error (false positive) are: the Bonferroni correction, the Holm-Bonferroni method, or the Dunn-Šidák correction. These tools, however, are known to increase false negatives (Armstrong, 2014). Regardless, if we would use a Dunn-Šidák correction to adjust for 3 multiple comparisons per topic, then the minimum *P* value for claiming a statistically significant result would not be .05 but instead something close to .017.

Nevertheless, the collected findings still support Hypothesis 1. As shown in Fig. 6 and Table 3, many *P* values are much less than .017.

## Footnotes

1 An *explainee* is a person receiving an explanation.

2 The word *explanandum* means “what has to be explained” in Latin.

3 This theory has been supported by various experiments and studies involving over 190 participants (Sovrano & Vitali, 2022a, b).

4 The explanatory space is the space of all possible bits of explanation about an explanandum. A more formal definition is given by Sovrano and Vitali (2022a).

5 The BVA is an administrative tribunal within the United States Department of Veterans Affairs that determines whether U.S. military veterans are entitled to claimed veterans’ benefits and services.

6 A legal memorandum is an organized document that summarizes relevant laws to support a conclusion on a particular legal issue.

7 Intelligent textbooks extend regular textbooks by integrating machine-manipulable knowledge (Wang et al., 2021).

8 The verbatim question is a question for which an answer can be identified in a related instructional text (i.e., source text) (Shimmei & Matsuda, 2022).

9 One of the first known philosophers to work on the concept of *explanation* was Aristotle with his *theory of causation* (Stein, 2011).

10 YAI4Hu stands for YAI for Humans. Examples of YAI for reinforcement learning agents are discussed by Sovrano et al. (2022).

11 We cannot release the textbook excerpts (Brostoff & Sinsheimer, 2013) because they are copyrighted.

12 The smallest unit of coherent text that expresses a complete or partial discourse relation.

13 A semantic formalism that represents the meaning of natural language sentences as rooted, directed, and labeled graphs.

14 A discourse relation identifies two EDUs: the first one is encoded in the question, the second one in the answer.

15 T5 is an encoder-decoder model based on the assumption that all Natural Language Processing problems can be converted in a text-to-text problem.

16 An epoch is one complete cycle through the entire training dataset.

17 Pertinence scores are numbers in [0, 1] computed by measuring the cosine similarity between vectorial representations of question and answer obtained through deep neural networks specialized for answer retrieval.

18 https://github.com/PrithivirajDamodaran/Gramformer

19 A question is said to be similar to another when its similarity score is above a given threshold *s*. Similarity scores are calculated like pertinence scores.

20 A typical *clause* consists of a subject and a syntactic predicate, the latter typically a verb phrase composed of a verb with any objects and other modifiers.

21 A nominal modifier is a noun (or noun phrase) functioning as a non-core (oblique) argument or adjunct.

22 Known as the “en_core_web_md” model, it boasts an accuracy score of 0.97. More details about this model can be found at https://spacy.io/models/en.

23 RDF is a standard model for data interchange on the Web (Allemang and Hendler, 2011).

24 A URI, or Uniform Resource Identifier, is a string of characters that provides a simple and extensible means for identifying a resource on the Internet.

25 RDFS, or RDF Schema, is a language used for creating vocabularies in the semantic web, allowing for the description of classes, properties, and their interrelationships.

26 We received explicit consent from the copyright holder to use excerpts of this textbook for our experiments and the related scientific publications.

27 https://www.law.cornell.edu

28 https://en.wikipedia.org/wiki/Law_of_the_United_States

29 https://search.usa.gov/search?affiliate=bvadecisions &sort_by= &query=PTSD &commit=Search

30 https://www.law.pitt.edu/academics/courses/catalog/5719
